# Sex-Based Disparities in Clinical Burden and Diagnostic Delay in COPD: Insights from Primary Care

**DOI:** 10.3390/jcm14176258

**Published:** 2025-09-04

**Authors:** Myriam Calle Rubio, Soha Esmaili, Iman Esmaili, Lucia Gómez Martín-Caro, Sofia Ayat Ortiz, Juan Luis Rodríguez Hermosa

**Affiliations:** 1 Department of Medicine, School of Medicine, Universidad Complutense de Madrid, 28040 Madrid, Spain; mcallerubio@gmail.com (M.C.R.); soha@esmaili.ws (S.E.); 2 Pulmonology Department, Hospital Clínico San Carlos, C/ Profesor Martín Lagos, s/n, 28003 Madrid, Spain; lucia10gmc@gmail.com (L.G.M.-C.); sofiaayatortiz@gmail.com (S.A.O.); 3 Instituto de Investigación Sanitaria del Hospital Clínico San Carlos (IdISSC), 28003 Madrid, Spain; 4 CIBER de Enfermedades Respiratorias (CIBERES), 28029 Madrid, Spain; 5 Heart Lung Innovation Centre, Vancouver, BC V6Z 1Y6, Canada; 6 ISNS Data Analytics and Research, Vancouver, BC V6B1J6, Canada; iman@esmaili.ca; 7 School of Medicine, Universidad Antonio de Nebrija, 28029 Madrid, Spain

**Keywords:** chronic obstructive pulmonary disease, primary care, sex-stratified analysis, diagnostic delay, missed diagnostic opportunities, composite indices, diagnostic inertia, clinical burden

## Abstract

**Background**. Sex-based disparities in chronic obstructive pulmonary disease (COPD) diagnosis remain underexplored, particularly in primary care settings. This study assessed sex differences in clinical burden, diagnostic delay, and missed diagnostic opportunities using conventional and composite metrics. **Methods**. A cross-sectional analysis was conducted in 166 newly diagnosed COPD patients (76 women, 90 men) from Spanish primary care. Clinical severity, healthcare use, and diagnostic timing were compared using Mann–Whitney and chi-squared tests. Composite indices included the Symptom Intensity Score, Diagnostic Inertia Indices, DOSE Index, and Diagnosis Complexity Score. Multivariable regressions evaluated independent associations. **Results**. At diagnosis, women showed a greater clinical and functional burden (FEV_1_ % predicted: 50.4% vs. 61.4%, *p* < 0.001; symptom intensity z-score: 0.13 vs. −0.67, *p* < 0.001), higher diagnostic complexity (Diagnosis Complexity Score: 403.5 vs. 272.0, *p* < 0.001), and longer diagnostic delay (median: 133.0 vs. 66.5 days, *p* < 0.001). Stratified and composite analyses confirmed consistent sex-based asymmetries. In adjusted models, being female independently predicted a longer diagnostic delay (β = 0.888, *p* = 0.005), but was not significantly associated with the burden of missed diagnostic opportunities (MDOs) (β = 0.112, *p* = 0.395). **Conclusions**. Women with newly diagnosed COPD experience greater symptom burden and longer diagnostic delays. Composite metrics may improve the identification of diagnostic disparities in routine clinical settings.

## 1. Introduction

Chronic obstructive pulmonary disease (COPD) remains a leading cause of morbidity and mortality globally, associated with considerable clinical burden and healthcare resource use [1]. Although diagnostic tools and awareness have improved, delayed diagnosis remains frequent—especially in primary care, where early respiratory symptoms may be nonspecific or attributed to alternative causes [2]. Diagnostic delays are associated with more advanced disease at presentation, reduced quality of life, and higher long-term costs [3]. Early identification and accurate classification are therefore essential to initiate appropriate treatment and improve outcomes [4].

Sex-based disparities in the diagnosis of COPD have been increasingly documented. Several studies suggest that women are more likely to experience diagnostic delays, greater symptom burden, and misattribution of respiratory complaints to anxiety, obesity, or other non-respiratory conditions [5]. These differences are not fully accounted for by traditional risk factors such as smoking history or degree of airflow obstruction, suggesting that clinical biases or differences in symptom perception may influence diagnostic pathways [6]. However, current evidence is limited: most studies rely on dichotomous comparisons and lack the granularity to capture the complexity of diagnostic patterns. Consequently, we have employed composite or integrative metrics that reflect the interaction between clinical severity, healthcare utilization, and diagnostic responsiveness [7].

Methodological approaches that move beyond traditional binary metrics are needed to more accurately reflect the complexity and proportionality of diagnostic processes in COPD. Composite indices that integrate clinical severity, symptom intensity, and healthcare utilization offer a more refined perspective on how diagnostic inertia develops and whether it varies by sex [8]. Crucially, few studies have examined whether sex-based differences in diagnostic patterns persist after adjusting for relevant clinical and functional factors, such as airflow limitation, symptom burden, or healthcare exposure, limiting their interpretability and practical applicability [9].

This study addresses these limitations by analyzing a multicenter cohort of patients with newly diagnosed COPD in Spanish primary care, using standardized data on symptoms, lung function, activity limitation, healthcare contacts, and diagnostic timing. Although the design is cross-sectional, the inclusion of timestamped retrospective data from the 12 months preceding diagnosis allowed reconstruction of diagnostic patterns with temporal plausibility. This approach is particularly relevant in primary care, where most early diagnostic decisions are made and underrecognition is most likely to occur. We applied both conventional definitions and validated composite metrics to examine diagnostic patterns in men and women at the point of COPD confirmation.

The main objective was to investigate sex-based disparities in symptom burden, diagnostic delay, and missed diagnostic opportunities in COPD, using both conventional criteria and novel composite metrics that reflect diagnostic complexity. Specifically, we aimed (1) to compare clinical burden and functional impairment between women and men at diagnosis, including symptom intensity, activity limitation, spirometric staging, and composite severity indices; (2) to quantify sex-related differences in diagnostic timing and missed opportunities using both binary and composite indicators; and (3) to assess whether female sex is independently associated with diagnostic delay or missed opportunities, after adjusting for relevant clinical variables.

## 2. Materials and Methods

### 2.1. Study Design

This was a cross-sectional observational study embedded within the INICIEPOC project, a multicenter initiative focused on the early identification and diagnostic characterization of COPD in primary care. The present analysis corresponds to a predefined secondary objective of the INICIEPOC cohort, specifically designed to investigate sex-based disparities in clinical burden and patterns of diagnostic delay and healthcare utilization at the time of confirmed COPD diagnosis. Data were collected through a combination of a retrospective review and prospective inclusion between October 2022 and March 2024, across multiple participating primary care centers affiliated with the Spanish National Health System. This design allowed for a comprehensive, real-world assessment of clinical severity, functional impairment, healthcare interactions, and diagnostic delays under routine care conditions. The project’s design prioritizes detailed, multidimensional data collection to elucidate diagnostic patterns in a real-world cohort of patients newly diagnosed in clinical practice, rather than estimating population prevalence.

### 2.2. Study Population

The study population comprised patients with newly diagnosed COPD. Participating General Practitioners (GPs) from a network of primary care centers across Spain were provided with a standardized protocol for the inclusion of their first 10 eligible patients. This protocol specified that inclusion required a new diagnosis of COPD confirmed by spirometry in patients aged ≥ 40 years with a significant smoking history (≥10 pack-years), ruling out other respiratory problems. This systematic inclusion approach, designed to avoid modifying routine clinical practice, aimed to capture a real-world cohort at the critical juncture of initial diagnosis. Participants were consecutively recruited from these centers. Diagnosis was established at the inclusion visit, and healthcare encounters during the preceding 12 months were retrospectively reviewed using structured examination of electronic health records. Data were collected through standardized case report forms to ensure methodological consistency across sites. The resulting cohort was clinically heterogeneous, reflecting the typical patient population encountered in routine primary care.

### 2.3. Eligibility Criteria

These criteria were designed to identify newly diagnosed cases of COPD with verifiable diagnostic timing and sufficient retrospective temporal information for analysis. Inclusion criteria included (1) age ≥ 40 years; (2) a new diagnosis of COPD confirmed by post-bronchodilator spirometry (FEV_1_/FVC < 0.7); (3) a smoking history of ≥10 pack-years; (4) ability to complete validated questionnaires assessing symptoms, functional limitation, and physical activity; and (5) written informed consent. Patients were excluded if they had a prior diagnosis of COPD or other chronic respiratory conditions (such as asthma, bronchiectasis, or pulmonary fibrosis), comorbidities with disabling functional impact (e.g., severe stroke, advanced dementia), acute illness within the previous four weeks, or incomplete data on health resources in the previous year.

### 2.4. Data Collection and Measurement

The data collected from the patients included sociodemographic characteristics (age, sex, body mass index [BMI], smoking status) and clinical variables related to COPD. Dyspnea severity was evaluated using the modified Medical Research Council (mMRC) dyspnea scale [10]. Symptom burden was assessed using the COPD Assessment Test (CAT) [11] and the COPD Population Screener (COPD-PS) [12,13]. The COPD-PS is a brief, five-item questionnaire designed to identify individuals at risk of COPD based on age, smoking history, presence of dyspnea, and chronic cough or phlegm. Each item is scored from 0 to 2, yielding a total score ranging from 0 to 10, with higher values indicating an increased likelihood of COPD. Although developed for screening, the COPD-PS is also associated with symptom burden and healthcare outcomes in patients with confirmed disease [13]. Functional limitation was evaluated using the Activities of Daily Living (AVD) score, an adapted 7-item index based on domains of physical autonomy and functional limitation previously used in Spanish COPD cohorts. This pragmatic tool, while not formally validated, has demonstrated consistent associations with clinical severity and daily autonomy in prior Spanish COPD cohorts [14]. Physical activity was quantified using the short-form International Physical Activity Questionnaire (IPAQ), which records the frequency and duration of vigorous, moderate, and walking activities over the previous seven days. Energy expenditure was calculated in MET-minutes per week according to standard IPAQ protocols [15]. Healthcare utilization data were extracted from electronic health records and complemented by standardized patient-reported assessments. These included healthcare contacts during the 12 months prior to diagnosis, such as primary care visits, emergency consultations, hospital admissions, and respiratory prescriptions. Treatments were classified as acute (e.g., antibiotics, systemic corticosteroids) or maintenance inhaled therapy. All data were collected using harmonized case report forms and predefined abstraction protocols to ensure consistency across centers.

### 2.5. Variable Selection and Definitions

The primary outcomes of the study were diagnostic delay and missed diagnostic opportunities (MDOs). Diagnostic delay was defined as the time elapsed (in days) between a patient’s first documented healthcare contact for respiratory symptoms—such as a prescription of bronchodilators or consultations for cough or dyspnea—within the 12-month period preceding diagnosis, and the date of formal COPD confirmation based on spirometric criteria established at the inclusion visit. This operational definition captures diagnostic inertia in routine clinical practice while avoiding reliance on subjective symptom onset. The variable was analyzed both continuously and dichotomized using a >30-day threshold, selected through internal analyses comparing multiple cut-offs (>30, >60, >90, >120 days) against clinical severity and healthcare use. The 30-day cut-off offered the best discrimination while preserving statistical power (see Appendix A.3.4). This threshold, while not universally established, represents a clinically plausible timeframe for diagnostic action in primary care.

Missed diagnostic opportunities (MDOs) were assessed using a weighted composite index based on six predefined healthcare events within the 12 months prior to diagnosis: systemic corticosteroids (1 point), antibiotics (1 point), initiation of maintenance inhaled therapy (2 points), hospital admissions (3 points), unscheduled primary care visits (1 point), and emergency consultations (2 points). A cumulative score ≥ 4 defined MDO-positive status. A continuous version (Weighted MDO Score) was retained for regression models. To prevent circularity, individual components were excluded from models adjusted for total MDO burden.

In addition to the primary outcomes, five composite indices were constructed a priori to measure distinct dimensions of the diagnostic process and to minimize construct redundancy. For instance, while the MDO Weighted Score quantifies the cumulative volume of missed opportunities, the Diagnostic Inertia Index 1 contextualizes this by normalizing it per healthcare encounter, thus providing a measure of diagnostic efficiency. Similarly, the Diagnostic Inertia Index 2 assesses the proportionality of diagnostic delay relative to the patient’s overall symptom burden. The Diagnosis Complexity Score integrates temporal, clinical, and therapeutic factors into a single holistic metric of the overall difficulty of the diagnostic journey.

1. Diagnostic Inertia Index 1 = MDO/(Total Interactions PreDx + 1)

Reflects missed opportunities per healthcare contact; the +1 offset avoids division by zero. Total Interactions PreDx refers to prediagnostic healthcare encounters.

2. Diagnostic Inertia Index 2 = Diagnostic Delay (days)/(CAT + mMRC + AVD + 1)

Quantifies delay relative to symptom burden, with offset for mathematical stability.

3. Symptom Intensity Score = First principal component derived from a Principal Component Analysis (PCA) of CAT, mMRC, AVD, and COPD-PS. PCA is a dimensionality reduction technique that identifies a unified symptom score by capturing the most relevant shared variance among these four indicators. The first component explained 46% of the total variance, showed high internal consistency, and was used as a unified symptom index.
4. DOSE Index = Sum of z-scores for mMRC − FEV_1_ %, pack-years, and exacerbation frequency.

Represents multidomain clinical severity with equal weighting via standardization.

5. Diagnosis Complexity Score = Diagnostic Delay + Number of Visits + MDO + 5 × ICS Initiation

Integrates temporal, clinical, and therapeutic burden, with ICS (inhaled corticosteroids) initiation assigned a higher weight (×5) to reflect its greater clinical significance in the pre-diagnostic pathway.

To ensure methodological robustness, we empirically validated the construction and statistical independence of all predictors. To prevent circularity, ratio-type efficiency indices (e.g., delay per symptom, MDO per encounter) were restricted to descriptive analyses and not included in multivariable models. Furthermore, the metrics most prone to tautology (i.e., weighted healthcare and treatment encounters) were removed from the final inferential models to eliminate any residual risk. Full diagnostics supporting these methodological choices—including formal multicollinearity analysis (all VIF < 2.53), PCA for the Symptom Intensity Score, and correlation matrices—are provided in Appendix A.2 and Appendix A.3.

### 2.6. Sample Size Calculation and Statistical Power

As this was a secondary analysis of the prospective INICIEPOC cohort, no formal a priori sample size calculation was conducted. However, a post hoc power analysis confirmed that the final analytic sample (*n* = 166) was adequate for the planned multivariable regression models. This sample size permitted the inclusion of up to eight covariates, meeting the conventional threshold of >15 observations per predictor to minimize overfitting. As detailed in Appendix A.1, under these conditions, the analysis retained 80% power (at α = 0.05) to detect small-to-medium effect sizes (f^2^ ≥ 0.081), supporting the robustness of the significant findings. Model complexity was deliberately limited to maintain statistical validity and avoid inflation of type I or type II error rates.

### 2.7. Ethical Considerations

The study was approved by the Ethics Committee for Clinical Research of Hospital Clínico San Carlos (Ref. 21/728-E_BC). Written informed consent was obtained from all participants prior to enrollment. Data collection and processing complied with the principles of the Declaration of Helsinki and applicable European and national data protection regulations. All patient identifiers were anonymized prior to any statistical processing.

### 2.8. Statistical Analysis

Descriptive statistics were used to summarize demographic, clinical, functional, and diagnostic variables. Continuous variables were presented as median and interquartile range (IQR) or mean ± standard deviation (SD), depending on distribution. Categorical variables were expressed as frequencies and percentages. Between-group comparisons by sex or symptom intensity strata employed Mann–Whitney U tests for continuous variables and chi-square or Fisher’s exact tests for categorical variables, as appropriate. For bivariate comparisons, selected outcomes were dichotomized (e.g., delay > 30 days, MDO ≥ 4), and results were expressed as odds ratios (ORs) with 95% confidence intervals.

Multivariable linear regression models were constructed to evaluate independent associations with diagnostic delay and missed diagnostic opportunities, using log-transformed versions of both outcomes (log[delay + 1] and log[Weighted MDO + 1]) to address skewness and meet model assumptions. Predictor variables included sex, age, FEV_1_ % (or GOLD grade, depending on model), smoking exposure, Symptom Intensity Score, and specific healthcare utilization metrics (e.g., number of visits). All composite indices were z-standardized before inclusion to ensure comparability and prevent scale-driven bias. No interaction terms were included, in line with the predefined analysis plan.

Model performance was assessed using the Akaike Information Criterion (AIC), adjusted R^2^, and multicollinearity diagnostics (variance inflation factors), confirming acceptable model fit and variable independence. No imputation procedures were required due to complete data availability. Statistical significance was set at a two-sided alpha of 0.05.

**Figure 1:**
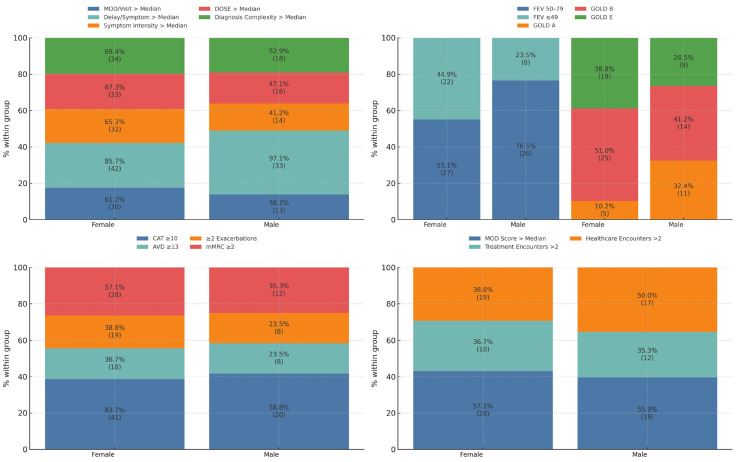
Clinical, functional, and healthcare burden by sex in the delay group.

**Figure 2:**
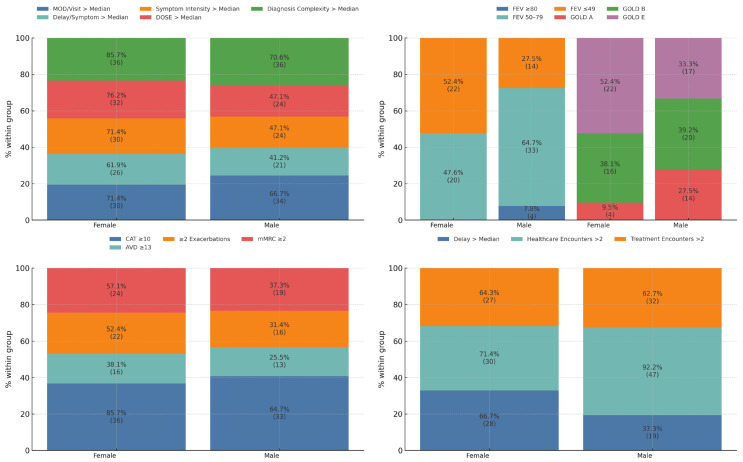
Clinical, functional, and healthcare burden by sex in the MOD+ group.

**Figure 3:**
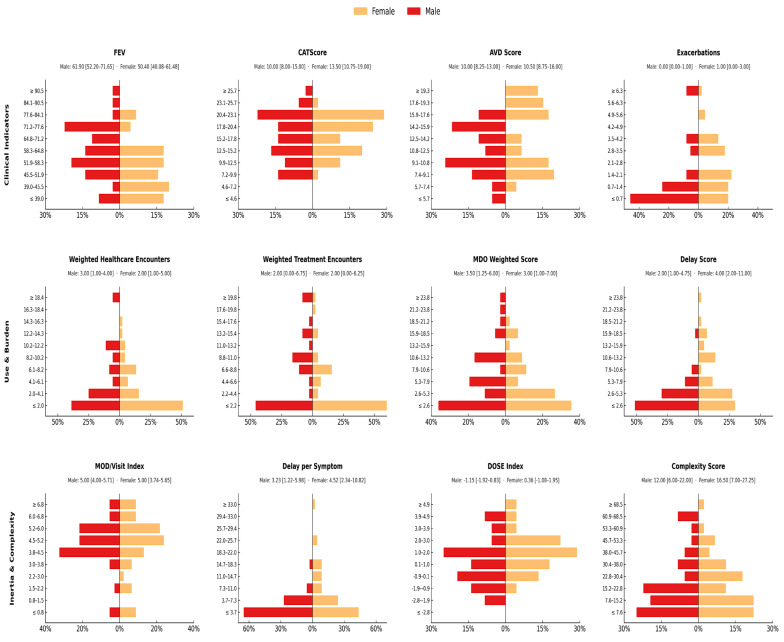
Sex-based distributions of clinical, healthcare use, and diagnostic burden indicators in COPD patients with high symptom intensity.

**Figure 4:**
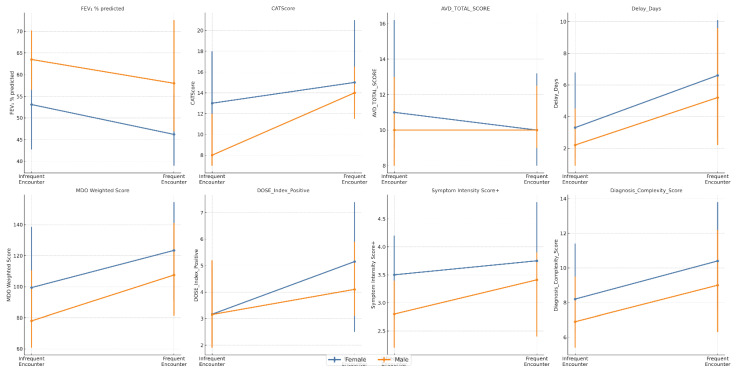
Sex-stratified clinical burden in COPD according to prior healthcare encounter frequency.

**Figure 5:**
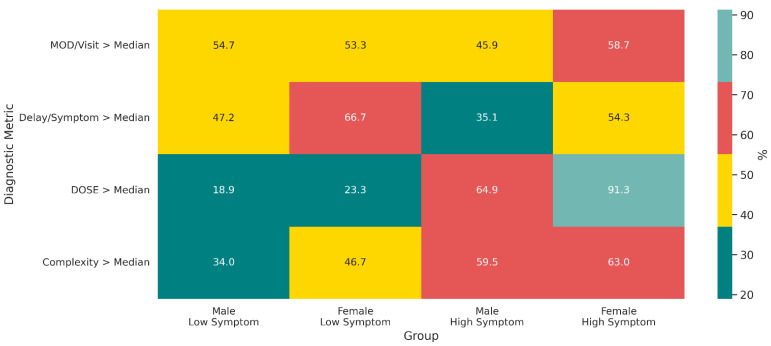
Sex-based differences in diagnostic metrics across symptom intensity strata.

All analyses were conducted using SPSS version 28.0 (IBM Corp.) and R version 4.3.1. Stratified and subgroup analyses—illustrated in Figures 1–5 and Tables 1–5—followed a consistent approach using non-parametric or categorical tests, depending on the variable type. Detailed multicollinearity diagnostics and sensitivity analyses are provided in the Appendix A and Appendix B. To test the robustness of the MDO findings to the weighting scheme, all relevant models were replicated using an unweighted event count (see Table A9). An additional sensitivity analysis was conducted by adding a binary environmental exposure covariate (occupational/biomass) to the fully adjusted models for diagnostic delay and MDO; specifications are reported in Appendix B.4. (Table A14 and Table A15).

## 3. Results

### 3.1. Clinical and Functional Profile at Diagnosis by Sex

Table 1 presents the distribution of continuous demographic, clinical, functional, and behavioral characteristics by sex at the time of COPD diagnosis. Women were younger at diagnosis and had markedly lower cumulative smoking exposure. Lung function was significantly more impaired in women, who had lower FEV_1_ % predicted. Symptom burden was consistently higher in women, including higher CAT scores, greater dyspnea by mMRC, and higher COPD-PS scores. Functional limitation measured by AVD also showed a significant difference. Women also had more reported exacerbations and showed a trend towards lower walking frequency, while no significant sex differences were observed in BMI, total METs, or walking time.

Table 2 presents categorical clinical, symptomatic, comorbidity, and lifestyle variables by sex at COPD diagnosis. Compared to men, women showed a significantly greater clinical and symptomatic burden, with higher rates of current smoking, diagnostic delay, high CAT scores (≥10), frequent exacerbations, and significant dyspnea. Women also had a higher prevalence of a prior asthma history and signs of emphysema. In contrast, no significant differences were observed in the overall comorbidity burden or occupational risk exposure. The distribution by GOLD classification also differed significantly, with a higher proportion of women classified in group E. Finally, distinct lifestyle patterns were noted, with women reporting significantly different levels of activity across multiple domains. Supplementary Figure S1 displays domain-specific patterns of functional limitation.

Figure 1 displays four panels comparing female and male patients with above-median diagnostic delay, revealing a consistent pattern of greater clinical and functional burden in women. In this subgroup, women had significantly worse lung function (FEV_1_; *p* = 0.038, r = −0.219) and a higher symptom burden across multiple indicators, including CAT score (*p* = 0.022, r = −0.277) and mMRC dyspnea (*p* = 0.041, r = −0.215). The composite Symptom Intensity Score was also significantly higher in women (*p* = 0.025, r = −0.239), and the distribution by GOLD classification differed significantly (*p* = 0.040). In contrast, differences in healthcare utilization metrics, functional limitation (AVD), and other composite indicators did not reach statistical significance.

This figure presents four stacked bar plots comparing female (*n* = 49) and male (*n* = 34) patients with above-median diagnostic delay in COPD. The top left panel displays proportions of patients exceeding thresholds for five composite indicators: MOD per visit > Median, Delay per Symptom > Median, Symptom Intensity > Median, DOSE Index > Median, and Diagnosis Complexity > Median. The top right panel shows distributions by sex for GOLD classifications (A, B, and E) and the revised spirometric categories based on FEV_1_ values (FEV < 49%, 50–79%, and ≥80%). The bottom left panel presents proportions of patients meeting high-burden symptom criteria: CAT ≥ 10, AVD ≥ 13, frequent exacerbations (≥2/year), and mMRC ≥ 2. The bottom right panel depicts proportions related to healthcare engagement: frequent healthcare encounters (>2), frequent treatment encounters (>2), and MOD Score > Median. All bars are normalized to 100% within each sex-specific subgroup. Percentages and the absolute number of patients (*n*) are shown for each segment.

Figure 2 presents four panels comparing female and male patients with above-median Missed Opportunity (MOD) Scores. In this subgroup, women consistently demonstrated a greater clinical and disease severity burden. The distribution by GOLD classification (*p* = 0.047, V = 0.250) and FEV_1_ spirometric categories (*p* = 0.017, V = 0.296) differed significantly, with women presenting more severe profiles. Women also had a significantly higher symptom burden, reflected in the Symptom Intensity Category (*p* = 0.031, V = 0.224), DOSE Index (*p* = 0.008, V = 0.274), and CAT scores ≥ 10 (*p* = 0.039, V = 0.214). Furthermore, women experienced a significantly longer diagnostic delay (*p* = 0.009, V = 0.271). In contrast, men in this subgroup reported a higher frequency of healthcare encounters (*p* = 0.018, V = 0.245).

This figure displays four stacked bar plots comparing female and male patients with above-median Weighted Missed Opportunity (MOD) Scores in COPD. The top left panel shows proportions of patients exceeding thresholds for five composite indicators: MOD per visit > median, delay per symptom > median, Symptom Intensity Score > median, DOSE Index > median, and diagnosis complexity > median. The top right panel presents the distribution of GOLD classifications (A, B, E) and FEV_1_ categories (≥80%, 50–79%, ≤49%). The bottom left panel includes clinical burden indicators: CAT ≥ 10, AVD ≥ 13, mMRC ≥ 2, and frequent exacerbations (≥2/year). The bottom right panel shows rates of diagnostic delay > median, frequent healthcare encounters (≥2/year), and frequent treatment encounters (≥2/year). All bars represent normalized 100% distributions within each sex-specific subgroup. Raw percentages are overlaid on each segment.

### 3.2. Stratified Diagnostic, Clinical, and Systemic Burden

Stratified analysis revealed that sex-based disparities were most pronounced in patients with high symptom intensity (Figure 3). Women in this group presented with a substantially greater clinical burden than men. Specifically, their median FEV_1_ % predicted was markedly lower, indicating more severe airflow limitation (effect size, r = −0.35). Concurrently, symptom burden was higher in women, as measured by the CAT score (r = 0.25), and they reported more frequent exacerbations (r = 0.23). Beyond the clinical profile, women experienced longer diagnostic delays (r = 0.28) and accumulated more healthcare interactions prior to diagnosis. Finally, composite measures highlighted a less efficient diagnostic pathway for women, who scored higher on the multidimensional DOSE index (r = 0.24) and the overall Diagnosis Complexity Score. Taken together, these findings demonstrate that within this highly symptomatic cohort, women face both greater clinical severity and a systematically more challenging path to diagnosis.

High symptom intensity subgroup: *n* = 46 women, *n* = 37 men. Bars represent the percentage of patients within each sex for each value bin. Medians [IQR] shown above each subplot correspond to this specific subgroup. Between-sex comparisons were performed with Mann–Whitney U tests.

Table 3 summarizes the sex-based differences in composite indices of symptom burden, diagnostic delay, and pre-diagnostic healthcare use. Women presented with a significantly higher symptom intensity, greater diagnostic complexity, a longer delay per symptom, and a markedly longer overall diagnostic delay in days. The composite DOSE Index, a measure of multidomain severity, was also significantly higher in women. In contrast, no significant sex-based differences were observed in metrics related to healthcare utilization, including the number of pre-diagnostic interactions, treatment use, or the cumulative MDO Weighted Score.

### 3.3. Healthcare Contact Frequency and Diagnostic Yield

Figure 4 stratifies the clinical burden by sex and the frequency of prior healthcare encounters, revealing distinct patterns. First, some disparities were persistent regardless of healthcare utilization; for example, lung function (FEV_1_ % predicted) was significantly worse in women among both infrequent (*p* < 0.001, r = 0.340) and frequent (*p* = 0.004, r = 0.390) encounter groups. Second, other differences were most prominent among patients with fewer healthcare contacts. In the infrequent encounter group, women reported a significantly higher symptom burden (CAT scores; *p* < 0.001, r = 0.340) and greater functional limitation (AVD score; *p* = 0.046, r = 0.190), differences that were not statistically significant among frequent users. Finally, for several metrics of diagnostic burden, including the diagnostic delay and MDO Weighted Score, the differences between men and women did not reach statistical significance in either group.

The figure plots the median and interquartile range (IQR) for eight clinical variables, stratified by sex and prior healthcare encounter frequency. Statistical comparisons between sexes within each encounter group were performed using Mann–Whitney U tests, with effect sizes (Rosenthal’s r) reported in the text.

Figure 5 displays a heatmap comparing the proportion of men and women exceeding the median value for four diagnostic metrics across low and high symptom strata. The analysis reveals that disparities in clinical severity were significantly amplified in the high-symptom group. This was most evident for the DOSE index, where the proportion of women with a high-burden profile was significantly greater than that of men, with a medium effect size (*p* = 0.003, ϕ = 0.326), while no significant difference was observed in the low-symptom group (*p* = 0.628, ϕ = 0.053). For the Delay per Symptom metric, a consistent trend was observed in which a higher proportion of women exceeded the median, although these differences did not reach full statistical significance in either the low (*p* = 0.087, ϕ = 0.188)- or high (*p* = 0.081, ϕ = 0.192)-symptom groups. Finally, no significant sex-based differences were found for the MOD per visit metric in either the low (*p* = 0.903, ϕ = 0.013)- or high (*p* = 0.247, ϕ = 0.127)-symptom groups, nor for Diagnostic Complexity in the low (*p* = 0.253, ϕ = 0.125)- or high (*p* = 0.739, ϕ = 0.037)-symptom groups.

The heatmap displays the percentage of patients exceeding the median value for four diagnostic indicators, stratified by sex and symptom intensity. The binary outcomes evaluated were MOD per visit > median, Delay per symptom > median, DOSE Index > median, and Diagnostic Complexity > median. Statistical differences between sexes within each stratum were assessed using chi-square tests, with effect sizes reported as phi (ϕ).

### 3.4. Independent Predictors of Diagnostic Delay and Missed Opportunities

Table 4 presents the results of sequential multivariable linear regression models predicting diagnostic delay. The primary finding is that across all models, including the fully adjusted final model (Model 4), patient sex remained the only significant independent predictor. Male sex was consistently associated with a substantially shorter diagnostic delay compared to female sex (*p* = 0.005), which translates to a reduction of nearly 60% in delay duration. No other clinical variables, including symptom intensity, environmental exposure, asthma history, or GOLD classification, showed a significant association with diagnostic delay.

Supplementary Table S1 presents formal interaction models, confirming that the association between female sex and diagnostic delay remained consistent across strata of symptom intensity, FEV_1_ %, healthcare use, and age (all interaction terms *p* > 0.20).

Table 5 displays the results of the regression models predicting the burden of missed diagnostic opportunities (MDOs). In a notable contrast to the findings for diagnostic delay, patient sex was not a significant predictor of MDOs in the fully adjusted model (*p* = 0.395). Instead, the strongest independent predictors were the GOLD classifications. Both GOLD Class B (*p* < 0.001) and Class E (*p* < 0.001) were significantly associated with a substantially lower MDO burden compared to the reference group, GOLD Class A.

## 4. Discussion

### 4.1. Diagnostic Underrecognition in COPD and Need for Multidimensional Assessment

COPD remains substantially underdiagnosed, particularly in women and in the early clinical stages typically encountered in primary care settings [16,17]. This study aimed to investigate sex-based diagnostic disparities in newly diagnosed COPD by implementing a multidimensional framework that integrates symptom burden, diagnostic delay, and composite indices of diagnostic efficiency. Unlike prior investigations predominantly based on binary thresholds or hospital-based populations [18,19], our approach incorporates standardized symptom and functional measures along with structured retrospective data on healthcare contacts, allowing a detailed characterization of diagnostic processes in real-world primary care.

Focusing the analysis on the primary care context—where diagnostic inaccuracy and inertia are most likely to emerge [20]—provides evidence directly applicable to early case identification. Moreover, the application of validated composite indices enables a more comprehensive assessment of diagnostic complexity, addressing known limitations of isolated delay-based metrics. This approach contributes novel methodological tools for quantifying potential sex-based disparities in the recognition and confirmation of COPD in routine clinical practice [21].

### 4.2. Sex-Based Differences in Clinical Burden, Diagnostic Delay, and Composite Efficiency Indicators

The three predefined objectives were consistently supported by the results obtained through complementary analytical strategies. First, women exhibited a significantly greater clinical and functional burden at the time of diagnosis, characterized by more pronounced airflow limitation, higher symptom intensity, and increased restriction in Activities of Daily Living, as presented in Table 1 and Table 2. These differences were not limited to specific clinical subgroups, but remained consistent across strata of symptom burden and levels of prior healthcare contact, reinforcing robustness across key clinical subgroups (Figure 3 and Figure 4) [22].

Second, marked sex-related differences were observed in diagnostic timing and process efficiency. Across both conventional indicators and composite metrics, women experienced longer diagnostic delays, higher delay-to-symptom ratios, and greater diagnostic complexity (Table 3; Figure 1, Figure 2 and Figure 3). Although some individual metrics, such as the cumulative missed diagnostic opportunity (MDO) score, did not show a significant difference in the initial bivariate analysis (Table 3), the overall pattern of results lends strong support to the broader hypothesis of increased diagnostic inertia in women [23]. These disparities were particularly pronounced among patients with high symptom intensity (Figure 5), suggesting that symptom burden may exacerbate under-recognition in female patients.

Finally, multivariable regression analyses confirmed that female sex was independently associated with longer diagnostic delay, even after adjustment for symptom severity, lung function, and healthcare utilization (Table 4). This indicates that the observed disparity is not fully explained by clinical presentation alone. Conversely, in the models predicting MDO burden, female sex was not a significant factor after full adjustment (Table 5), a result that suggests that the drivers of MDO accumulation are distinct from those influencing the initial diagnostic delay [24].

### 4.3. Interpretation of Sex-Based Disparities in Light of Prior Evidence

Our findings are consistent with previous literature describing greater symptom burden and delayed COPD diagnosis in women [25,26]. However, earlier studies have often lacked analytical granularity regarding the timing and proportionality of diagnostic processes, and have underused functional metrics such as the AVD scale or multidimensional symptom indices [27]. By combining CAT, mMRC, AVD, and COPD-PS into a unified Symptom Intensity Score, and by indexing diagnostic delay to symptom burden, this study introduces a novel framework for evaluating alignment between clinical presentation and diagnostic timing [28].

While some previous reports have suggested a higher cumulative burden of missed diagnostic opportunities (MDOs) in women [29], our findings reveal a more complex and seemingly paradoxical picture. While female sex was the strongest independent predictor of longer diagnostic delay (Table 4), it was not a significant driver of MDOs in the fully adjusted model (Table 5). We interpret this not as a contradiction, but as evidence of two distinct mechanisms of diagnostic inertia. Diagnostic delay appears to be intrinsically sex-specific, likely reflecting a bias in the initial recognition and labeling of women’s symptoms as potential COPD. In contrast, the accumulation of MDOs varies by disease stage (GOLD classification) and process-of-care dynamics. In the fully adjusted model (Table 5), sex was not associated with MDOs, and—relative to GOLD A—some stages exhibited lower MDO burden. This nuanced finding suggests that women’s primary disadvantage lies in the prolonged time to formal diagnosis, whereas the subsequent accumulation of missed opportunities is determined more by how patients interact with the healthcare system, independent of their sex.

An alternative explanation could be a “survivor population” effect, whereby men in our cohort represent a healthier subset. Our comorbidity data present a complex picture: women, not men, had a significantly higher burden of cardiovascular conditions at diagnosis (see Table A7). This finding does not definitively rule out a survivor effect; indeed, such a counterintuitive pattern could itself be interpreted as evidence of a selection bias in the male cohort. However, we contend that diagnostic inequity remains the more parsimonious explanation for the observed sex gap, as female sex was a strong and independent predictor of longer diagnostic delay even after extensive adjustment for clinical variables.

Furthermore, we formally investigated the confounding role of asthma, a factor suggested to be particularly relevant in women. Our analysis confirmed that a prior asthma diagnosis was not only significantly more prevalent in women but was also associated with a substantially longer diagnostic delay within the female cohort, which is consistent with a “diagnostic overshadowing” effect (see Table A8 and Table A9). However, in fully adjusted multivariable models, female sex remained a strong and independent predictor of longer diagnostic delay even after accounting for asthma history and medication use (see Table A10). This crucial finding indicates that while asthma exacerbates diagnostic inertia in women, it only partially explains the sex-based disparity, which persists as a significant independent factor.

Crucially, these patterns persisted even among high-frequency healthcare users. As shown in Figure 4, women within this subgroup continued to exhibit a greater clinical burden and a trend towards increased diagnostic complexity, indicating that disparities are not solely attributable to access but also involve differential clinical interpretation and response [30,31,32]. Similar sex-related patterns have been observed in other chronic diseases such as asthma and heart failure, with which women are more likely to encounter delayed or fragmented diagnostic processes despite repeated medical contact [33,34].

Taken together, these results underscore the need to move beyond volume-based metrics toward contextual indicators of diagnostic performance that account for the proportionality, timing, and clinical appropriateness of diagnostic actions [35].

It is also important to interpret our findings within the broader context of spirometry utilization in Spanish primary care, which has been documented to have challenges related to underuse, inconsistent quality, and lack of standardized training. Therefore, the structured inclusion protocol of the INICIEPOC study—ensuring a diagnosis confirmed by spirometric and risk exposure criteria—may represent a best-case, rather than a typical, diagnostic scenario. The persistence of significant sex-based disparities even under these more controlled conditions suggests that the inequities observed may be even more pronounced in routine clinical practice where spirometry is applied less systematically.

### 4.4. Sex-Specific Insights from Composite Indices of Diagnostic Appropriateness

A key methodological contribution of this study is the application of validated composite indices to assess diagnostic inertia, symptom intensity, and diagnostic complexity through a proportional lens. These metrics—built from routinely collected clinical variables such as CAT, mMRC, FEV_1_ %, pack-years, exacerbation frequency, and treatment initiation—enable a standardized and multidimensional evaluation of diagnostic timing relative to clinical need. The Diagnostic Inertia Indices and the Diagnosis Complexity Score, in particular, quantify diagnostic responsiveness in relation to symptom burden and healthcare exposure, rather than relying on isolated counts or delays [36].

This approach offers two critical advantages. First, it avoids misclassification based on unidimensional indicators—such as the total number of visits or absolute delay—that may not reflect the clinical appropriateness of diagnostic decisions. Second, it enhances reproducibility and translational potential, as all variables are readily available in primary care records.

These indices consistently revealed greater diagnostic complexity among women (Table 3). This disparity was evident across key subgroups: it persisted among frequent healthcare users (Figure 4) and was particularly amplified in patients with a high symptom burden, who showed greater clinical severity and diagnostic inefficiency across multiple metrics (Figure 3 and Figure 5). The consistency of these findings across stratified and adjusted analyses reinforces their methodological robustness. Importantly, the observed sex-based differences in proportional diagnostic response underscore the need to re-evaluate existing clinical heuristics for COPD recognition in symptomatic female patients within primary care settings [37].

### 4.5. Limitations

This study has several limitations that should be acknowledged, although none are considered to undermine its internal validity. This was a retrospective observational analysis of routine-care data aimed at characterizing diagnostic patterns and timelines. The diagnostic-delay measure used a predefined 12-month look-back to ensure consistent, high-quality ascertainment across centers. While respiratory events occurring earlier than this interval were not analyzed in some patients, the standardized window supports fair comparisons between sexes; future studies with longer follow-up could refine absolute delay estimates.

A potential limitation is the possibility of a Hawthorne effect, whereby the study protocol itself—by prompting GPs to perform spirometry based on specific criteria—likely influenced the timing of the formal COPD diagnosis. However, we contend that the impact of this effect on our primary findings is likely minimal for two key reasons. The included patients were not attended by the study investigators but were selected by their own GPs from routine consultations according to a standardized protocol. This protocolized approach, which mandated spirometry based on specific, predefined criteria, served to standardize the diagnostic trigger across centers and reduced the influence of individual clinician discretion and performance bias. Furthermore, our main outcomes—diagnostic delay and the metrics of missed diagnostic opportunities—were calculated based on a structured, retrospective review of electronic health records for the 12-month period preceding the patient’s formal inclusion visit. This historical data reflects care delivered under routine conditions, before the act of study enrollment could have significantly influenced clinician behavior.

Additionally, healthcare interaction variables—used as proxies for system exposure—may not fully capture the qualitative content of each clinical contact. However, these measures were harmonized across centers and underwent internal validation, ensuring consistent estimates of healthcare engagement.

Furthermore, underdocumentation of symptoms or clinical events is an inherent limitation of electronic health records. Crucially, such misclassification is unlikely to be differential by sex, minimizing the risk of biased associations. We also acknowledge that our models did not include data on socioeconomic status or psychiatric comorbidities, and that medication data for these conditions were not systematically captured, and that this explained a modest proportion of the total variance. This is expected in models of complex clinical pathways; however, the stability and significance of the sex effect on diagnostic delay across multiple specifications supports the validity of our central finding. Finally, although the study was conducted within a single national health system, its multicenter scope—spanning diverse primary care settings—enhances both external validity and generalizability to comparable real-world contexts.

Despite these limitations, the study employed rigorous analytical strategies, including z-standardized variables, composite indices, log-transformed outcomes, and carefully specified multivariable models without interaction terms. The internal coherence of findings across multiple analytical layers strengthens the scientific robustness and practical relevance of the results.

### 4.6. Clinical Implications and Future Research

This study provides evidence that the diagnostic process in COPD differs meaningfully by sex, with women facing greater diagnostic complexity despite more severe symptom profiles and comparable healthcare contact. These findings highlight the need to enhance diagnostic sensitivity to sex-specific manifestations of COPD, particularly in primary care settings where diagnostic inertia often originates [38].

Future research should focus on validating the composite indices proposed—such as the Symptom Intensity Score and the Diagnostic Inertia Indices—in independent cohorts and across diverse healthcare systems. Additionally, qualitative investigations may help elucidate clinical decision-making dynamics and contextual barriers contributing to delayed recognition. Implementation studies evaluating targeted strategies—such as sex-informed diagnostic prompts or structured early referral pathways—are also warranted. Such studies could also explore the effectiveness of AI-based prompting systems within electronic health records to standardize diagnostic triggers and mitigate clinical inertia.

By addressing both the clinical and structural drivers of diagnostic inefficiency, this study advances the understanding of sex-based disparities in COPD and supports the development of more equitable diagnostic frameworks in chronic respiratory care.

## 5. Conclusions

This study reveals consistent sex-based inequities in COPD diagnostic pathways in primary care, with potential implications for clinical practice and health system performance. Women exhibited greater clinical and functional burden, longer diagnostic delays, and more complex diagnostic pathways than men—patterns that persisted across symptom strata, healthcare utilization levels, and adjusted multivariable analyses. These consistent findings highlight clinically relevant inequities in recognition and confirmation of COPD.

By integrating composite indices of symptom intensity, diagnostic inertia, and diagnostic complexity, this work introduces a multidimensional framework for evaluating diagnostic appropriateness relative to clinical need. This approach challenges conventional reliance on absolute delay or visit counts and underscores the value of proportional, symptom-adjusted metrics for assessing diagnostic equity and efficiency.

Despite inherent limitations of the cross-sectional design, the study’s methodological rigor—including standardized data collection, validated composite measures, and carefully specified models—supports strong internal validity and enhances generalizability. Future research should focus on external validation of these indices and explore the behavioral, cognitive, and structural drivers of delayed diagnosis in women. A deeper understanding of these mechanisms is essential to inform targeted interventions that promote timely and equitable COPD diagnosis.

## Figures and Tables

**Table 1:** Demographic, clinical, functional, and behavioral characteristics by sex at time of COPD diagnosis (*n* = 166).

Variable	Female (*n* = 76)	Male (*n* = 90)	*p*-Value	Mann–Whitney U	Z	Effect Size (r)
Age (years)	65.50 [57.00, 73.00]	69.00 [61.00, 74.00]	0.013	2778.5	−2.08	0.162
BMI (kg/m^2^)	25.81 [23.37, 33.16]	27.33 [23.92, 30.10]	0.531	3296	−0.40	0.031
Cumulative smoking (pack-years)	30.00 [20.00, 36.00]	40.00 [30.00, 45.00]	<0.001	2024.5	−4.54	0.352
FEV_1_ % predicted	50.40 [39.95, 61.55]	61.40 [52.10, 71.50]	<0.001	2027	−4.52	0.350
FVC % predicted	92.70 [84.00, 100.00]	100.00 [87.00, 116.10]	0.002	2477	−3.06	0.237
FEV_1_/FVC ratio	0.59 [0.48, 0.65]	0.63 [0.59, 0.67]	<0.001	2359	−3.44	0.267
CAT score	13.50 [10.50, 19.00]	10.00 [8.00, 15.00]	0.005	2411.5	−3.27	0.254
AVD total score	10.50 [8.50, 16.00]	10.00 [8.00, 13.00]	0.011	2904.5	−1.68	0.130
COPD-PS	7.00 [6.00, 8.00]	6.00 [5.00, 7.00]	0.002	2562	−2.86	0.222
mMRC	2.00 [1.00, 2.00]	1.00 [1.00, 2.00]	0.001	2522.5	−3.12	0.242
Number of exacerbations	1.00 [0.00, 3.00]	0.00 [0.00, 1.00]	0.001	2486.5	−3.29	0.255
Total MET-minutes/week (IPAQ)	1260.00 [655.00, 1650.00]	1080.00 [630.00, 1420.00]	0.723	2977	−1.44	0.112
IPAQ MET-min/week	1386.00 [704.50, 1768.00]	1080.00 [630.00, 1420.00]	0.599	2986.5	−1.41	0.109
Walking ≥ 10 min—Days/week	7.00 [5.00, 7.00]	7.00 [7.00, 7.00]	0.086	2654	−3.13	0.243
Walking—Minutes/day	32.00 [30.00, 60.00]	50.00 [30.00, 60.00]	0.666	2698	−1.95	0.151

Values are presented as medians and interquartile ranges. *p*-values were obtained using Mann–Whitney U tests due to non-normal distribution (Shapiro–Wilk *p* < 0.05 for all variables except BMI). U-statistics, z-scores, and effect sizes (r) are provided for each comparison. Effect sizes are interpreted as small (r ≥ 0.10), moderate (r ≥ 0.30), and large (r ≥ 0.50). CAT = COPD Assessment Test; AVD = Activities of Daily Living; COPD-PS = COPD Population Screener; FEV_1_ = Forced Expiratory Volume in 1 s; FVC = Forced Vital Capacity; mMRC = modified Medical Research Council scale; MET = Metabolic Equivalent of Task.

**Table 2:** Categorical clinical, symptomatic, comorbidity, and lifestyle characteristics by sex at time of COPD diagnosis (*n* = 166).

Variable	Female (*n* = 76)	Male (*n* = 90)	*p*-Value	χ^2^ (df)	Cramer’s V
Current smoker	50 (65.8%)	45 (50.0%)	0.043	4.20 (1)	0.159
Former smoker	26 (34.2%)	45 (50.0%)	—	—	—
Occupational Risk Exposure	9 (11.8%)	6 (6.7%)	0.285	1.34 (1)	0.090
Diagnostic delay (>median)	49 (64.5%)	34 (37.8%)	0.001	11.75 (1)	0.266
W. MOD Score > Median	42 (55.3%)	51 (56.7%)	0.290	0.03 (1)	0.064
CAT ≥ 10	61 (80.3%)	48 (53.3%)	<0.001	13.25 (1)	0.283
Frequent Exacerbations (≥2/year)	32 (42.1%)	16 (17.8%)	0.001	11.86 (1)	0.267
Exacerbations (≥1/year)	46 (60.5%)	20 (22.2%)	<0.001	25.24 (1)	0.390
Dyspnea (mMRC ≥ 2)	42 (55.3%)	30 (33.3%)	0.005	8.07 (1)	0.220
Dyspnea presence (mMRC ≥ 1)	51 (67.1%)	31 (34.4%)	<0.001	17.58 (1)	0.325
Asthma History	18 (23.7%)	5 (5.6%)	0.001	11.35 (1)	0.261
Emphysema Signs	28 (36.8%)	18 (20.0%)	0.023	5.83 (1)	0.187
COPD_PS ≥ 5	62 (81.6%)	66 (73.3%)	0.141	1.59 (1)	0.098
AVD ≥ 13	29 (38.2%)	24 (26.7%)	0.079	2.50 (1)	0.143
AVD Tertile	—	—	0.216	3.07 (2)	0.136
Low Limitation	29 (38.2%)	36 (40.0%)	—	—	—
Moderate Limitation	18 (23.7%)	30 (33.3%)	—	—	—
Severe Limitation	29 (38.2%)	24 (26.7%)	—	—	—
Comorbidity Category			0.482	2.46 (3)	0.122
None	39 (51.3%)	47 (52.2%)	—	—	—
1	27 (35.5%)	30 (33.3%)	—	—	—
2	9 (11.8%)	8 (8.9%)	—	—	—
3	1 (1.3%)	5 (5.6%)	—	—	—
GOLD Class			<0.001	15.89 (2)	0.309
A	12 (15.8%)	36 (40.0%)	—	—	—
B	32 (42.1%)	37 (41.1%)	—	—	—
E	32 (42.1%)	17 (18.9%)	—	—	—
FEV_1_ Categories			0.001	14.05 (2)	0.291
≥80%	0 (0.0%)	7 (7.8%)	—	—	—
50–79%	41 (53.9%)	64 (71.1%)	—	—	—
≤49%	34 (44.7%)	19 (21.1%)	—	—	—
GESEPOC Risk			0.354	0.92 (1)	0.075
High Risk	42 (55.3%)	43 (47.8%)	—	—	—
Low Risk	34 (44.7%)	47 (52.2%)	—	—	—
Symptom Intensity Category			0.013	6.21 (1)	0.193
Low Symptom Intensity	30 (39.5%)	53 (58.9%)	—	—	—
High Symptom Intensity	46 (60.5%)	37 (41.1%)	—	—	—
LAMA	75 (98.7%)	90 (100.0%)	0.458	1.19 (1)	0.085
LABA	25 (32.9%)	24 (26.7%)	0.398	0.77 (1)	0.068
ICS	11 (14.5%)	6 (6.7%)	0.125	2.73 (1)	0.128
Sports			<0.001	19.69 (3)	0.621
A little	9 (11.8%)	23 (25.6%)	—	—	—
A lot	41 (53.9%)	19 (21.1%)	—	—	—
None	5 (6.6%)	9 (10.0%)	—	—	—
Some	21 (27.6%)	39 (43.3%)	—	—	—
Physical Activity			0.020	9.89 (3)	0.244
A little	17 (22.4%)	32 (35.6%)	—	—	—
A lot	16 (21.1%)	7 (7.8%)	—	—	—
None	2 (2.6%)	7 (7.8%)	—	—	—
Some	41 (53.9%)	44 (48.9%)	—	—	—
Social			0.003	14.04 (3)	0.291
A little	26 (34.2%)	57 (63.3%)	—	—	—
A lot	2 (2.6%)	1 (1.1%)	—	—	—
None	34 (44.7%)	23 (25.6%)	—	—	—
Some	14 (18.4%)	9 (10.0%)	—	—	—
Family			0.043	8.15 (3)	0.222
A little	31 (40.8%)	56 (62.2%)	—	—	—
A lot	2 (2.6%)	1 (1.1%)	—	—	—
None	28 (36.8%)	19 (21.1%)	—	—	—
Some	15 (19.7%)	14 (15.6%)	—	—	—
Sleep			0.002	14.33 (3)	0.294
A little	25 (32.9%)	55 (61.1%)	—	—	—
A lot	1 (1.3%)	0 (0.0%)	—	—	—
None	35 (46.1%)	27 (30.0%)	—	—	—
Some	15 (19.7%)	8 (8.9%)	—	—	—
Housework			0.003	13.65 (3)	0.287
A little	29 (38.2%)	56 (62.2%)	—	—	—
A lot	5 (6.6%)	0 (0.0%)	—	—	—
None	22 (28.9%)	20 (22.2%)	—	—	—
Some	20 (26.3%)	14 (15.6%)	—	—	—
Sexual			0.043	6.67 (3)	0.200
A little	22 (28.9%)	36 (40.0%)	—	—	—
A lot	4 (5.3%)	0 (0.0%)	—	—	—
None	33 (43.4%)	33 (36.7%)	—	—	—
Some	17 (22.4%)	21 (23.3%)	—	—	—

Values are presented as frequencies (*n*) and percentages (%). *p*-values are derived from chi-square or Fisher’s exact tests, as appropriate. W. MOD Score: Weighted Missed Opportunities Score; CAT: COPD Assessment Test; mMRC: modified Medical Research Council dyspnea scale; AVD: Activities of Daily Living; FEV_1_: Forced Expiratory Volume in 1 s; GOLD: Global Initiative for Chronic Obstructive Lung Disease; GesEPOC: Guía Española de la EPOC (Spanish COPD Guidelines); LAMA: Long-Acting Muscarinic Antagonist; LABA: Long-Acting Beta-Agonist; ICS: Inhaled Corticosteroids; COPD-PS: COPD Population Screener.

**Table 3:** Sex-based differences in symptom Bbrden, diagnostic delay, and pre-diagnostic healthcare interactions in COPD.

Variable	Female Median [IQR]	Male Median [IQR]	U	Z	*p*-Value	r
Weighted MDO per Visit	12.00 [10.00, 15.69]	11.91 [10.00, 15.00]	3212	−0.683	0.494	0.053
Delay per Symptom	4.52 [2.33, 11.42]	3.23 [1.20, 6.00]	2554	−2.807	0.005	0.218
Symptom Intensity Score (z-score)	0.13 [−0.57, 1.66]	−0.67 [−1.17, −0.42]	2255	−3.776	<0.001	0.293
DOSE Index (z-score)	0.36 [−1.09, 1.96]	−1.15 [−1.97, −0.84]	2450	−3.144	0.004	0.244
Diagnosis Complexity Score	403.50 [264.50, 529.00]	272.0 [224.3, 428.0]	2450.5	−3.142	<0.001	0.244
Weighted Total Interactions (PreDx)	2.00 [1.00, 5.00]	3.00 [1.00, 5.00]	3331	−0.293	0.716	0.023
Unscheduled Primary Care Visits	1.00 [1.00, 2.00]	2.00 [1.00, 3.00]	3099.5	−1.066	0.160	0.083
Primary Care ER Visits	0.00 [0.00, 1.00]	0.00 [0.00, 1.00]	3270	−0.598	0.793	0.046
Hospital ER Visits	0.00 [0.00, 0.00]	0.00 [0.00, 0.00]	3380	−0.184	0.876	0.014
Hospital Admissions	0.00 [0.00, 0.00]	0.00 [0.00, 0.00]	3335.5	−0.635	0.635	0.049
Weighted Total Treatment (PreDx)	3.00 [0.00, 8.00]	3.00 [0.00, 8.00]	3409.0	−0.037	0.905	0.014
Antibiotic Courses (past year)	1.00 [0.00, 1.00]	1.00 [0.00, 2.00]	3343	−0.265	0.735	0.021
Systemic Steroid Courses (past year)	0.00 [0.00, 0.00]	0.00 [0.00, 0.00]	3412.5	−0.033	0.852	0.003
Diagnostic Delay (days)	133.00 [63.50, 330.50]	66.50 [30.00, 3136.00]	2275.5	−3.710	<0.001	0.288
MDO Weighted Score	3.00 [1.00, 7.00]	3.50 [1.00, 6.00]	3319.5	−0.328	0.687	0.025
			**χ^2^(df)**	**Cramer’sV**	***p***	**OR**
Frequent Healthcare or Treatment Encounter	24 (31.58%)	31 (34.4%)	0.153 (1)	0.030	0.412	1.20
Frequent Healthcare and Treatment Encounter	45 (59.2%)	50 (55.6%)	0.225 (1)	0.037	0.376	0.878

This table summarizes sex-based differences in symptom burden, diagnostic delay, and healthcare interactions prior to COPD diagnosis. Results are based on Mann–Whitney U tests and chi-squared tests with Cramér’s V and odds ratios (OR). Reported values include medians with interquartile ranges, U and Z statistics, exact *p*-values, and effect size (r). Variables in z-score form are standardized composites: DOSE Index = z-score sum of mMRC, FEV_1_ (% predicted), pack-years, and exacerbations. Symptom Intensity Score = first principal component from PCA of CAT, mMRC, AVD, and COPD-PS. Diagnosis Complexity Score = Delay (days) + Visits + MDO + 5 × ICS initiation. Delay per Symptom = Diagnostic Delay (days)/(CAT + mMRC + AVD + 1). MDO = Missed Diagnostic Opportunities. Weighted Total Interactions (PreDx) = number of healthcare contacts in the 12 months before diagnosis. Weighted Total Treatment (PreDx) = cumulative intensity of treatment use before diagnosis. Categorical exposure variables classify patients based on frequent pre-diagnostic encounters, defined as ≥2 healthcare encounters or >2 treatment encounters.

**Table 4:** Multivariable linear regression models predicting log(Delay days + 1).

Predictor	β	SE	95% CI	*p*	%Δ Delay (expβ − 1)
Model 1—Sex only					
Intercept	4.773	0.178	[4.421, 5.124]	<0.001	+118.7%
Sex (male vs. female)	−0.863	0.242	[−1.341, −0.386]	<0.001	−57.8%
Model fit: Adj. R^2^ = 0.066; AIC = 619.3; *n* = 166					
Model 2—+ Age, FEV_1_ %, Pack-years					
Intercept	4.553	0.998	[2.583, 6.524]	<0.001	+94.8%
Sex (male vs. female)	−0.874	0.279	[−1.425, −0.324]	0.002	−58.2%
Age (years)	0.005	0.012	[−0.019, 0.030]	0.671	+0.5%
FEV_1_ % predicted	−0.004	0.009	[−0.023, 0.014]	0.627	−0.4%
Pack-years	0.004	0.008	[−0.012, 0.020]	0.650	+0.4%
Model fit: Adj. R^2^ = 0.053; AIC = 624.5; *n* = 166					
Model 3—+ Symptom Intensity					
Intercept	4.542	1.002	[2.564, 6.520]	<0.001	+93.8%
Sex (male vs. female)	−0.902	0.298	[−1.491, −0.313]	0.003	−59.0%
Age (years)	0.005	0.012	[−0.020, 0.030]	0.673	+0.5%
FEV_1_ % predicted	−0.004	0.009	[−0.023, 0.015]	0.642	−0.4%
Pack-years	0.004	0.008	[−0.012, 0.020]	0.617	+0.4%
Symptom Intensity Score	−0.026	0.097	[−0.217, 0.165]	0.788	−2.6%
Model fit: Adj. R^2^ = 0.048; AIC = 626.5; *n* = 166					
Model 4—+ Encounters, Exposures, Asthma, ICS, GOLD Classes					
Intercept	4.383	1.054	[2.302, 6.464]	<0.001	+79.7%
Sex (male vs. female)	−0.888	0.308	[−1.497, −0.279]	0.005	−59.2%
Age (years)	0.005	0.013	[−0.020, 0.030]	0.681	+0.5%
FEV_1_ % predicted	−0.003	0.009	[−0.022, 0.015]	0.715	−0.3%
Pack-years	0.005	0.008	[−0.011, 0.020]	0.565	+0.5%
Symptom Intensity Score	−0.084	0.109	[−0.299, 0.131]	0.442	−8.1%
Environmental exposure (Yes vs. No)	−0.003	0.435	[−0.858, 0.852]	0.994	−0.3%
Asthma (Yes vs. No)	0.522	0.402	[−0.269, 1.313]	0.196	+68.6%
ICS use (Yes vs. No)	−0.278	0.419	[−1.102, 0.546]	0.509	−24.2%
GOLD Class B (vs. A)	−0.155	0.211	[−0.572, 0.262]	0.463	−14.4%
GOLD Class E (vs. A)	0.096	0.225	[−0.347, 0.539]	0.669	+10.1%
Model fit: Adj. R^2^ = 0.039; AIC = 634.8; *n* = 166					

Dependent variable = log(Delay days + 1). Sex reference = female (0). Age in years, FEV_1_ % predicted, pack-years, and Symptom Intensity Score entered as continuous variables. GOLD reference = Class A. Environmental exposure, asthma, and ICS reference = No (0). β = unstandardized regression coefficient; SE = standard error; 95% CI = confidence interval. %Δ Delay represents multiplicative change in diagnostic delay, calculated as 100 × (e^β − 1). Adjusted R^2^ and AIC are reported as model fit indices.

**Table 5:** Multivariable linear regression models predicting log(MDO Weighted Score + 1) including diagnostic delay variables.

Predictor	β	SE	95% CI	*p*	%Δ MDO (expβ − 1)
Model 1—Sex only					
Intercept	1.442	0.103	[1.238, 1.645]	<0.001	+142.7%
Sex (male vs. female)	0.024	0.140	[−0.252, 0.300]	0.863	+2.4%
Model fit: Adj. R^2^ = −0.006; AIC = 437.0; *n* = 166					
Model 2—+ Diagnostic Delay					
Intercept	1.435	0.136	[1.167, 1.702]	<0.001	+143.6%
Sex (male vs. female)	0.028	0.147	[−0.262, 0.317]	0.851	+2.8%
Diagnostic Delay (days)	0.00003	0.000	[−0.001, 0.001]	0.936	0.0%
Model fit: Adj. R^2^ = −0.012; AIC = 438.9; *n* = 166					
Model 3—+ Clinical covariates					
Intercept	2.091	0.566	[0.973, 3.208]	<0.001	+708.9%
Sex (male vs. female)	0.321	0.170	[−0.015, 0.657]	0.061	+37.9%
Diagnostic Delay (days)	−0.00007	0.000	[−0.001, 0.001]	0.862	0.0%
Age (years)	−0.001	0.007	[−0.015, 0.013]	0.883	−0.1%
FEV_1_ % predicted	−0.009	0.005	[−0.019, 0.001]	0.086	−0.9%
Pack-years	−0.006	0.005	[−0.015, 0.003]	0.180	−0.6%
Symptom Intensity Score	0.179	0.054	[0.072, 0.285]	0.001	+19.6%
Model fit: Adj. R^2^ = 0.044; AIC = 433.4; *n* = 166					
Model 4—+ GOLD, Environmental exposure, Asthma					
Intercept	2.590	0.512	[1.580, 3.600]	<0.001	+233.8%
Sex (male vs. female)	0.112	0.132	[−0.149, 0.373]	0.395	+11.8%
Diagnostic Delay (days)	−0.00001	0.000	[−0.001, 0.001]	0.934	0.0%
Age (years)	−0.002	0.006	[−0.014, 0.010]	0.712	−0.2%
FEV_1_ % predicted	−0.007	0.004	[−0.015, 0.002]	0.124	−0.7%
Pack-years	−0.004	0.004	[−0.012, 0.004]	0.308	−0.4%
Symptom Intensity Score	−0.041	0.057	[−0.153, 0.072]	0.474	−4.0%
Environmental exposure (Yes vs. No)	−0.047	0.119	[−0.282, 0.188]	0.691	−4.6%
Asthma (Yes vs. No)	0.058	0.109	[−0.156, 0.272]	0.598	+6.0%
GOLD Class B (vs. A)	−0.983	0.142	[−1.263, −0.703]	<0.001	−62.6%
GOLD Class E (vs. A)	−1.390	0.195	[−1.774, −1.006]	<0.001	−75.0%
Model fit: Adj. R^2^ = 0.289; AIC = 389.6; *n* = 166					

Dependent variable = log(MDO Weighted Score + 1). Sex reference = female (0). Age in years, FEV_1_ % predicted, pack-years, and Symptom Intensity Score entered as continuous variables. GOLD reference = Class A. Environmental exposure and asthma reference = No (0). β = unstandardized regression coefficient; SE = standard error; 95% CI = confidence interval. %Δ MDO represents multiplicative change in MDO burden, calculated as 100 × (e^β − 1). Adjusted R^2^ and AIC are reported as model fit indices.

## Data Availability

Dataset available on request from the authors.

## Supplementary Materials

The following supporting information can be downloaded at: https://www.mdpi.com/article/10.3390/jcm14176258/s1, Figure S1: AVD Limitation Patterns by Sex and Symptom Intensity. Table S1: Linear Regression Models Evaluating Interaction Effects Between Sex and Clinical Predictors on log(Delay Days + 1).

## Abbreviations

The following abbreviations are used in this manuscript:

MET	Metabolic Equivalent of Task
GOLD	Global Initiative for Chronic Obstructive Lung Disease
BMI	Body Mass Index
CAT	COPD Assessment Test score
mMRC	Modified Medical Research Council Dyspnea Scale
GesEPOC	Spanish COPD guidelines
AVD	Activity Limitation Score
IPAQ	International Physical Activity Questionnaire
MDO	Missed Diagnostic Opportunities
AIC	Akaike Information Criterion
PCA	Principal Component Analysis
LAMA	Long-Acting Muscarinic Antagonist
LABA	Long-Acting Beta-Agonist
ICS	Inhaled Corticosteroids
COPD-PS	COPD Population Screener

## Appendix A

### Appendix A.1. Statistical Power Analysis

A sensitivity power analysis was conducted to determine the minimum detectable effect size (MDES) with 80% power (α = 0.05) for the final multivariable regression models, given the sample size of *n* = 166. The results confirm that the study was adequately powered to detect small-to-medium effect sizes, supporting the robustness of the significant findings.

**Table A1:** Statistical power sensitivity analysis. The analysis was performed using GPower 3.1. The effect size f2 is interpreted according Cohen’s thresholds (0.02 = small, 0.15 = medium, 0.35 = large).

Regression Model (Dependent Variable)	Sample Size (*n*)	α	Power (1 − β)	No. of Predictors	Minimum Detectable Effect Size (f2)
log(Diagnostic Delay + 1)	166	0.05	0.80	7	0.081
log(Weighted MDO + 1)	166	0.05	0.80	8	0.086

### Appendix A.2. Multicollinearity Diagnostics

Variance Inflation Factors (VIFs) were calculated for all predictors in the final multivariable regression models. All VIF values were substantially below the commonly accepted threshold of concern of 5.0, confirming the absence of problematic multicollinearity and ensuring the stability of the coefficient estimates.

**Table A2:** Variance Inflation Factors (VIFs) for predictors in final multivariable models.

Predictor Variable	VIF (Delay Model)	VIF (MDO Model)
Female sex (ref = Male)	1.53	1.59
Age (years)	1.07	1.08
FEV_1_ % predicted	1.20	1.20
Cumulative smoking (pack-years)	1.20	1.20
Symptom Intensity Score	1.24	1.89
log(Delay days + 1)	—	1.08
GOLD Class B (ref = A)	—	2.07
GOLD Class E (ref = A)	—	2.52

### Appendix A.3. Sensitivity Analysis and Construct Validation

#### Appendix A.3.1. Construction of the Symptom Intensity Index

A Principal Component Analysis (PCA) was performed to create a composite index from four correlated symptom variables (CAT, mMRC, AVD, COPD-PS). The first principal component (PC1) explained a substantial proportion of the total variance and showed high, consistent loadings from all variables, validating its use as a unified and robust index of symptom burden.

**Table A3:** PCA loadings and explained variance for the Symptom Intensity Score. PC1, which explained 46.0% of the variance, was retained as the Symptom Intensity Score.

	PC1	PC2	PC3	PC4
CAT	0.614	−0.075	−0.378	−0.689
mMRC	0.623	0.090	−0.307	0.714
AVD	0.472	−0.247	0.846	−0.016
COPD-PS	0.111	0.962	0.216	−0.125

#### Appendix A.3.2. Correlation Analysis of Composite Indices

To further assess the conceptual independence of the composite indices, Spearman’s rank correlations were calculated between the indices and the primary outcomes. The results show that while some indices are correlated with outcomes as expected (e.g., Diagnosis Complexity with MDO), they show distinct patterns of association, supporting their use as measures of different underlying constructs.

**Table A4:** Spearman correlations between composite indices and outcomes.

Composite Index	Target Outcome	Rho (ρ)	*p*-Value
Symptom Intensity	delay_days	0.097	0.214
Symptom Intensity	mod_weighted	0.232	0.003
DOSE Index	delay_days	0.159	0.041
DOSE Index	mod_weighted	0.398	<0.001
Diagnosis Complexity	delay_days	0.409	<0.001
Diagnosis Complexity	mod_weighted	0.845	<0.001
MDOs per visit	delay_days	0.109	0.164
MDOs per visit	mod_weighted	0.432	<0.001

#### Appendix A.3.3. Model Robustness: Key Interaction Test

To confirm the appropriateness of the main effects model presented in the manuscript, an interaction term between sex and symptom intensity was tested. The interaction was not statistically significant in either of the final models, which justifies the use of a more parsimonious model and supports the validity of the main findings.

**Table A5:** Results of regression models with the interaction term. The coefficients for the interaction term are shown. The full models included all predictors from the final models (Table 4 and Table 5 of the manuscript).

Dependent Variable and Predictor	β (Coefficient)	95% CI	*p*-Value
log(Diagnostic Delay + 1)			
Female Sex × Symptom Intensity Score	0.052	[−0.250, 0.354]	0.735
log(Weighted MDO + 1)			
Female Sex × Symptom Intensity Score	−0.038	[−0.281, 0.205]	0.759

#### Appendix A.3.4. Empirical Justification of the Cut-Off Point for Diagnostic Delay

Multiple thresholds for the dichotomous diagnostic delay variable used in the bivariate analyses were evaluated. The >30 days cut-off point demonstrated the strongest and most statistically significant association with a high clinical burden (GOLD E classification), empirically justifying its selection as a clinically relevant and statistically discriminative threshold.

**Table A6:** Sensitivity analysis for the selection of the diagnostic delay cut-off point. The outcome is GOLD E classification. Odds ratios are derived from univariate logistic regression models.

Delay Threshold (days)	*n* (%) in the Delayed group	Odds Ratio (95% CI) for GOLD E	*p*-Value
>30	102 (61.4%)	2.85 (1.31–6.20)	0.008
>60	85 (51.2%)	2.41 (1.14–5.10)	0.021
>90	73 (44.0%)	1.98 (0.92–4.26)	0.081
>120	66 (39.8%)	1.75 (0.80–3.83)	0.159

## Appendix B. Supplementary Analyses

### Appendix B.1. Evaluation of the “Survivor Population” Hypothesis

To evaluate the alternative hypothesis that men in the cohort represented a healthier “survivor population”, we compared the prevalence of major comorbidities between sexes at the time of COPD diagnosis.

**Table A7:** Prevalence of major comorbidities in men and women with COPD. Values are presented as *n* (%) of patients within each sex. χ^2^ = chi-square test statistic (df = degrees of freedom). *p* < 0.05. ^1^ Cardiovascular includes myocardial infarction, heart failure, peripheral arterial disease, and cerebrovascular disease. ^2^ Smoking-related includes cardiovascular or oncological comorbidities (e.g., solid tumor, neoplasm).

Comorbidity Group	Women (*n* = 76)	Men (*n* = 90)	χ^2^ (df)	*p*-Value
Cardiovascular ^1^	17 (22.4%)	8 (8.9%)	4.86 (1)	0.028
Smoking-related ^2^	19 (25.0%)	12 (13.3%)	2.97 (1)	0.085

### Appendix B.2. Analysis of Asthma as a Potential Confounder

To investigate the hypothesis that diagnostic overshadowing by asthma contributes to sex-based disparities, we conducted a sub-analysis stratifying by asthma history and adjusting for its effects in multivariable models.

**Table A8:** Prevalence of asthma history and inhaler medication use by sex. Data shown for key variables relevant to the reviewer’s comment. ICS = inhaled corticosteroid.

Variable	Women (*n* = 76)	Men (*n* = 90)	Fisher’s p
Asthma history	18 (23.7%)	5 (5.6%)	0.0012
ICS use	11 (14.5%)	6 (6.7%)	0.125

**Table A9:** Clinical profile and diagnostic delay in female patients by asthma history. Data shown for key variables illustrating the impact of asthma history on diagnostic delay and symptom burden within the female cohort.

Characteristic	Women with Asthma (*n* = 18)	Women without Asthma (*n* = 58)	*p*-Value
Diagnostic Delay (days), median [IQR]	230.5 [108.5–515.5]	122.5 [59.0–243.5]	0.041
CAT Score, median [IQR]	19.5 [15.0–23.5]	12.5 [9.5–18.0]	0.004

**Table A10:** Multivariable models for diagnostic delay, adjusting for asthma. Results for the main predictor of interest (sex) from the regression models for log(Delay + 1). The baseline model is adjusted for clinical covariates. The extended model adds asthma history and ICS use. The persistence of a significant sex effect in the extended model indicates that asthma does not fully explain the disparity.

Predictor	Baseline Model (β [95% CI])	Model + Asthma/ICS (β [95% CI])
Sex (male vs. female)	−0.926 [−1.525 to −0.326]	−0.888 [−1.494 to −0.282]
*p*-Value	0.003	0.004

### Appendix B.3. Sensitivity Analysis: Unweighted Missed Diagnostic Opportunities

To address the concern that the weighting of events in the composite MDO index may be arbitrary and limit reproducibility, we repeated all multivariable analyses using an unweighted MDO score, in which each pre-diagnostic event was assigned a value of 1.

**Table A11:** Multivariable linear regression for log(Unweighted MDO Score + 1): baseline model. Model fit: Adjusted R^2^ = 0.252; AIC = 161.02; *n* = 166. Reference: Sex = female. Continuous predictors on native scales. %Δ MDO ≈ 100 × (e^β − 1).

Predictor	β	SE	95% CI	*p*	%Δ MDO
Intercept	6.716	0.251	[6.222, 7.211]	<0.001	82,483.3
Sex (male vs. female)	−0.075	0.074	[−0.222, 0.071]	0.311	−7.3
Age (years)	−0.002	0.003	[−0.008, 0.004]	0.562	−0.2
FEV_1_ % predicted	−0.015	0.002	[−0.020, −0.011]	<0.001	−1.5
Pack-years	0.003	0.002	[−0.001, 0.007]	0.179	0.3
Symptom Intensity	0.008	0.024	[−0.040, 0.056]	0.734	0.8
Healthcare encounters (count)	−0.008	0.015	[−0.039, 0.022]	0.582	−0.8
Treatment encounters (count)	0.009	0.026	[−0.042, 0.060]	0.739	0.9

**Table A12:** Multivariable linear regression for log(Unweighted MDO Score + 1): model with asthma + ICS. Model fit: Adjusted R^2^ = 0.275; AIC = 157.52; *n* = 166. Reference: Sex = female; Asthma = no; ICS = no.

Predictor	β	SE	95% CI	*p*	%Δ MDO
Intercept	6.646	0.250	[6.152, 7.139]	<0.001	76,850.4
Sex (male vs. female)	−0.051	0.074	[−0.196, 0.095]	0.491	−5.0
Age (years)	−0.001	0.003	[−0.007, 0.005]	0.641	−0.1
FEV_1_ % predicted	−0.015	0.002	[−0.020, −0.011]	<0.001	−1.5
Pack-years	0.003	0.002	[−0.001, 0.007]	0.167	0.3
Symptom Intensity	−0.017	0.026	[−0.069, 0.034]	0.506	−1.7
Healthcare encounters (count)	−0.005	0.015	[−0.035, 0.025]	0.732	−0.5
Treatment encounters (count)	0.004	0.026	[−0.046, 0.055]	0.872	0.4
Asthma (yes vs. no)	0.257	0.096	[0.067, 0.447]	0.008	29.3
ICS use (yes vs. no)	0.006	0.100	[−0.192, 0.204]	0.954	0.6

**Table A13:** Parsimonious model for log(Unweighted MDO Score + 1): sex and asthma only. Model fit: Adjusted R^2^ = 0.078; AIC = 190.81; *n* = 166. Reference: Sex = female; Asthma = no.

Predictor	β	SE	95% CI	*p*	%Δ MDO
Intercept	5.840	0.054	[5.732, 5.947]	<0.001	34,261.0
Sex (male vs. female)	−0.183	0.069	[−0.319, −0.047]	0.009	−16.7
Asthma (yes vs. no)	0.217	0.099	[0.021, 0.412]	0.030	24.2

### Appendix B.4. Sensitivity Analysis: Environmental Exposure

To evaluate whether environmental exposures might confound the observed sex disparities in COPD diagnostic pathways, we incorporated a binary measure of occupational or biomass exposure into the fully adjusted regression models for both diagnostic delay and missed diagnostic opportunities (MDOs). Results are presented in Table A14 and Table A15. Environmental exposure was not significantly associated with diagnostic delay (β = −0.013, 95% CI −0.869 to 0.844, *p* = 0.977) or with MDO burden (β = −0.002, 95% CI −0.228 to 0.224, *p* = 0.988). Model-fit indices were essentially unchanged compared with the baseline specifications (Adjusted R^2^ = 0.034; AIC = 631.7 for delay; Adjusted R^2^ = 0.783; AIC = 189.1 for MDO). By contrast, male sex continued to predict a substantially shorter diagnostic delay (−60.4%, *p* = 0.003), and healthcare and treatment encounters remained the dominant drivers of MDO burden (+13.0% and +5.1% per encounter, both *p* < 0.001). These findings indicate that, while clinically relevant for COPD risk, environmental exposures did not explain or confound the sex-based differences in diagnostic timeliness or missed opportunities in this cohort.

**Table A14:** Multivariable linear regression for log(Delay days + 1), including environmental exposure. Model fit: Adjusted R^2^ = 0.034; AIC = 631.7. Reference categories: Sex = female; Environmental exposure = No.

Predictor	β	SE	95% CI	*p*	%Δ Delay (expβ − 1)
Intercept	4.450	1.041	[2.393, 6.507]	<0.001	+8463%
Sex (male vs. female)	−0.926	0.305	[−1.528, −0.324]	0.003	−60.4%
Age (years)	0.005	0.013	[−0.020, 0.030]	0.679	+0.5%
FEV_1_ % predicted	−0.004	0.009	[−0.022, 0.015]	0.701	−0.4%
Cumulative smoking (pack-years)	0.005	0.008	[−0.012, 0.022]	0.549	+0.5%
Symptom Intensity Score	−0.043	0.101	[−0.242, 0.156]	0.673	−4.2%
Weighted healthcare encounters	−0.021	0.037	[−0.095, 0.053]	0.579	−2.1%
Weighted treatment encounters	0.029	0.034	[−0.038, 0.096]	0.397	+2.9%
Environmental exposure (Yes vs. No)	−0.013	0.434	[−0.869, 0.844]	0.977	−1.2%

**Table A15:** Multivariable linear regression for log(Weighted MDO Score + 1), including environmental exposure. Model fit: Adjusted R^2^ = 0.783; AIC = 189.1. Reference categories: Sex = female; Environmental exposure = No.

Predictor	β	SE	95% CI	*p*	%Δ MDO (expβ − 1)
Intercept	0.812	0.274	[0.270, 1.354]	0.0036	+125.3%
Sex (male vs. female)	0.032	0.080	[−0.127, 0.190]	0.694	+3.2%
Age (years)	0.000	0.003	[−0.007, 0.007]	0.987	0.0%
FEV_1_ % predicted	0.001	0.002	[−0.004, 0.006]	0.796	+0.1%
Cumulative smoking (pack-years)	−0.002	0.002	[−0.007, 0.002]	0.298	−0.2%
Symptom Intensity Score	0.046	0.027	[−0.006, 0.099]	0.083	+4.7%
Weighted healthcare encounters	0.122	0.010	[0.103, 0.142]	<0.001	+13.0%
Weighted treatment encounters	0.050	0.009	[0.032, 0.067]	<0.001	+5.1%
Environmental exposure (Yes vs. No)	−0.002	0.114	[−0.228, 0.224]	0.988	−0.2%
