# Supplementary material for: Sex-Based Disparities in Clinical Burden and Diagnostic Delay in COPD: Insights from Primary Care
Source: J Clin Med. 2025 Sep 4;14(17):6258. doi: 10.3390/jcm14176258 (PMC12429159; doi:10.3390/jcm14176258)
Supplement: Supplementary file 1 [file jcm-14-06258-s001.zip › jcm-3826777-supplementary.pdf]

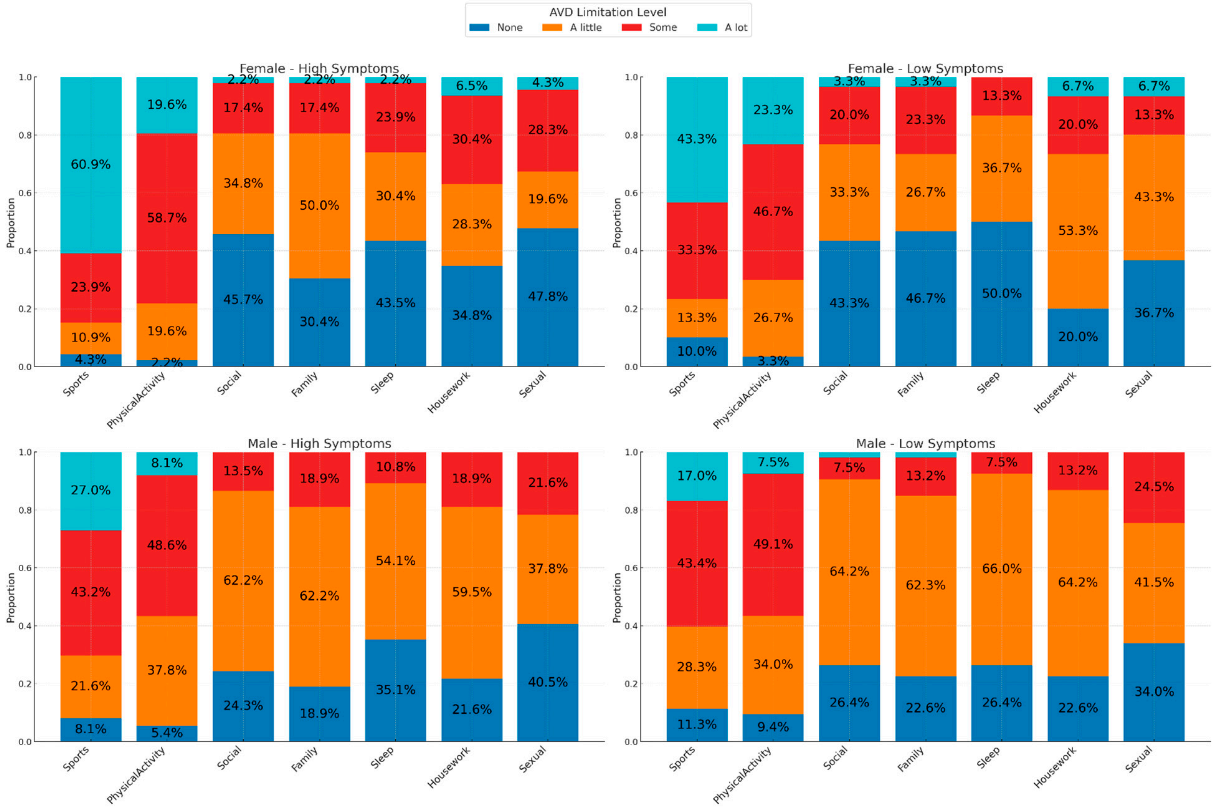

Supplementary Figure 1. AVD Limitation Patterns by Sex and Symptom Intensity.

AVD = Activities of Daily Living. Limitation levels include “None”, “A little”, “Some”, and “A lot”. Group comparisons reflect contrast between high vs. low symptom intensity within each sex, across each domain. OR = Odds Ratio; RR = Risk Ratio; χ^2 p-value = p-value from Chi-square test; h = Cohen’s h effect size (absolute value interpreted as small ≥ 0.20, medium ≥ 0.50, large ≥ 0.80). None of the comparisons reached statistical significance at the $p < 0.05$ level.

Supplementary Figure 1 presents the domain-specific distributions of AVD limitation severity, comparing high-symptom to low-symptom patients within each sex. Overall, the odds of reporting severe limitation were not statistically significantly different between symptom strata for most domains. For females, the comparisons were as follows: sports (OR = 1.70, p = 0.556), physical activity (OR = 1.54, p = 0.588), social (OR = 0.80, p = 0.916), family (OR = 0.67, p = 0.657), sleep (OR = 2.29, p = 0.296), and housework (OR = 1.61, p = 0.494). For males, the results were: sports (OR = 1.55, p = 0.459), physical activity (OR = 1.01, p = 1.000), social (OR = 1.50, p = 0.791), family (OR = 1.31, p = 0.848), sleep (OR = 1.48, p = 0.874), housework (OR = 1.53, p = 0.660), and sexual functioning (OR = 0.85, p = 0.946).

Interaction Term	β (SE)	t	p-value	95% CI for β	Adjusted R^2	AIC
Model 1: Sex × Symptom Intensity	0.029 (0.186)	0.152	.879	[–0.339, 0.396]	0.055	623.25
Model 2: Sex × FEV_1 % predicted	–0.021 (0.018)	–1.179	.243	[–0.057, 0.014]	0.065	621.59
Model 3: Sex × Healthcare Encounters	–0.022 (0.054)	–0.412	.682	[–0.128, 0.084]	0.056	623.13
Model 4: Sex × Age	–0.003 (0.021)	–0.134	.894	[–0.045, 0.039]	0.057	622.99

Supplementary Table 1. Linear Regression Models Evaluating Interaction Effects Between Sex and Clinical Predictors on log(Delay Days + 1). All models include the main effects of sex (reference: female), the interaction term, and the respective continuous covariate (Symptom Intensity Score, FEV_1 %, Healthcare Encounters, or Age). Dependent variable: log-transformed diagnostic delay in days. CI = Confidence Interval; SE = Standard Error; AIC = Akaike Information Criterion.

Supplementary Table 1 presents four linear regression models testing for interaction effects between patient sex and key clinical covariates on diagnostic delay. As shown, none of the tested interaction terms—sex × symptom intensity, sex × FEV_1 %, sex × healthcare encounters, or sex × age—reached statistical significance (all $p > 0.20$).
